# Direct observation of electric field-induced magnetism in a molecular magnet

**DOI:** 10.1038/s41598-023-29840-1

**Published:** 2023-02-16

**Authors:** M. Lewkowitz, J. Adams, N. S. Sullivan, Ping Wang, M. Shatruk, V. Zapf, Ali Sirusi Arvij

**Affiliations:** 1 Department of Physics, University of Florida, Florida, 32611 USA; 2 Department of Chemistry and Biochemistry, Florida State University, Florida, 32306 USA; 3 Los Alamos National Laboratory, Los Alamos, NM 87545 USA; 4 School of Science, Mathematics and Engineering, San Juan College, Farmington, NM 87402 USA

**Keywords:** Materials science, Physics

## Abstract

We report the direct observation of an electrically-induced magnetic susceptibility in the molecular nano- magnet [Fe_3_O(O_2_CPh)_6_(py)_3_]ClO_4_*·*py, an Fe_3_ trimer. This magnetoelectric effect results from the breaking of spatial inversion symmetry due to the spin configurations of the antiferromagnetic trimer. Both static and very low frequency electric fields were used. Fractional changes of the magnetic susceptibility of 11 ppb$\pm 2$ per kVm^-1^ for the temperature range 8*.*5 < *T* < 13*.*5 K were observed for applied electric fields up to 62 kV m^*−*1^. The changes in susceptibility were measured using a tunnel diode oscillator operating at liquid helium temperatures while the sample is held at a higher regulated temperature.

## Introduction

There is currently great interest in identifying simple molecular magnets that have quantum states that can be manipulated by external stimuli. Molecular magnets for which the magnetic and electric moments are coupled via a magnetoelectric (ME) effect^1–7^ are of particular interest both for developing an understanding of the underlying physics of the coupling mechanism, and for applications where an electric field can change the spin state. Developments in this area may open the frontier to a possible tunable quantum bit^8,9^.

Traditionally ME effects have been studied in inorganic oxides and/or materials with long-range magnetic and/or electric order. In paramagnetic non-interacting molecules, a different mechanism for a ME coupling exists, where the local spin configuration of each molecule can break inversion symmetry and create an electric dipole. For example, Dzyaloshinski-Moriya (DM) interactions^10,11^ can produce magnetic spin states in triangles that are non-centro-symmetric and polar. Plokhov et al.^12^ have shown that chirality can be induced in rare-earth spin clusters to result, in some cases, in electric dipoles.

**Figure 1:**
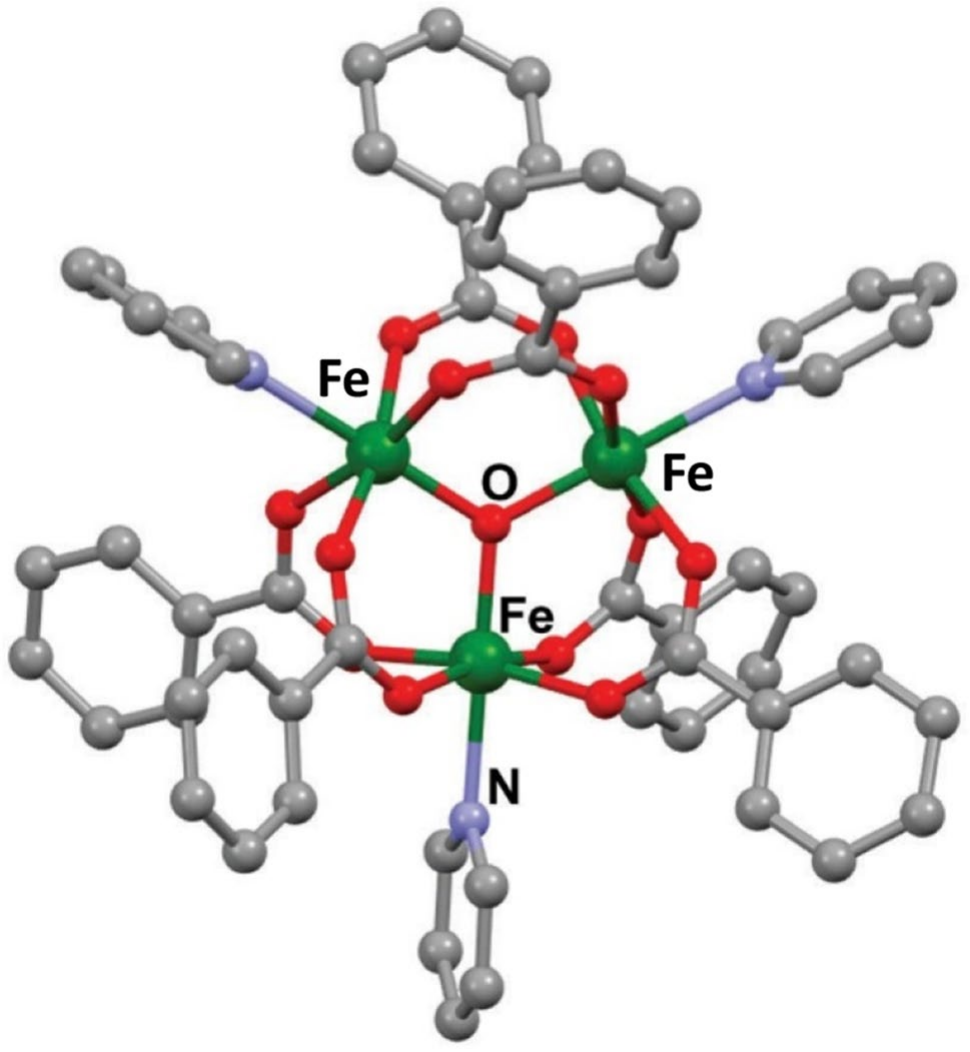
(Color online) Structure of the Fe_3_ carboxylate trimer as reported by Sowrey et al.^14^. Created from the Cambridge Crystallographic Data Base (entry code QOPLUC).

We have studied frustrated spin triangles with anti-ferromagnetic couplings that possess spin chirality as a result of the combined effect of frustrated exchange interactions and a Dzyaloshinski- Moriya interaction. Trif et al.^2^ and Bulaevskii et al.^13^ have proposed that the chirality eigen-value could be used as a qubit. These systems have a spin chirality that can be probed as the magnetic state is varied over a wide temperature range. Sowrey et al.^14^ have pointed out that the trinuclear oxo-centered carboxylate bridged complexes of the the general formula [M_3_^III^O(O_2_CR)_6_L_3_]^+^, and in particular [Fe_3_O(O_2_CPh)_6_(py)_3_]ClO_4_*·*py have the desirable symmetry, with half of the Fe^3+^ ions forming an isosceles triangle, and the other half of the ions forming a scalene triangle as shown in Fig.1. The molecules can be static or undergoing dynamic pseudo-rotations.

Indirect observation of an ME effect in the Fe_3_ trimer, [Fe_3_O(O_2_CPh_6_)(py)_3_]ClO_4_*·*py, has been reported by Boudalis et al.^15^. The amplitudes of the electron paramagnetic resonance (EPR) absorption lineshapes were modified by a few percent for static electric fields up to 10^9^ V m^*−*1^ but no displacements of the EPR line position were reported. George et al.^16^ have reported a ME effect for the antiferromagnetic wheel Cr_7_Mn in studies of the electron spin resonance (ESR) echo. In this paper we report direct observation of the ME coupling for a small electric field in crystalline samples of the aforementioned Fe_3_ cluster. There have been a few observations of ME effects already reported for molecular magnets^15,16^, but we report here what we believe is the first direct low frequency measurement.

## Experimental methods

The sample of Fe_3_ consisted of one mm-sized crystal and two smaller crystals (< 0.5 mm) glued together with Apiezon N-grease^17^ with the c-axis aligned parallel to the RF magnetic field of the oscillator. Two electrodes inside the RF coil provided an electric field perpendicular to the c-axis. The filling factor for the samples was of the order of 2%. The N-grease also served to thermalize the crystals to the regulated thermal block. N-grease by itself does not have a high thermal conductivity (typically 100 mW/Km at 4 K^18^, but it readily fills the micropores of adjoining surfaces and is thus excellent for thermal contact to the surface of a crystal at low temperatures. Furthermore, the N-grease does not lower the electronic quality factor (Q) of the resonant circuit.

**Figure 2:**
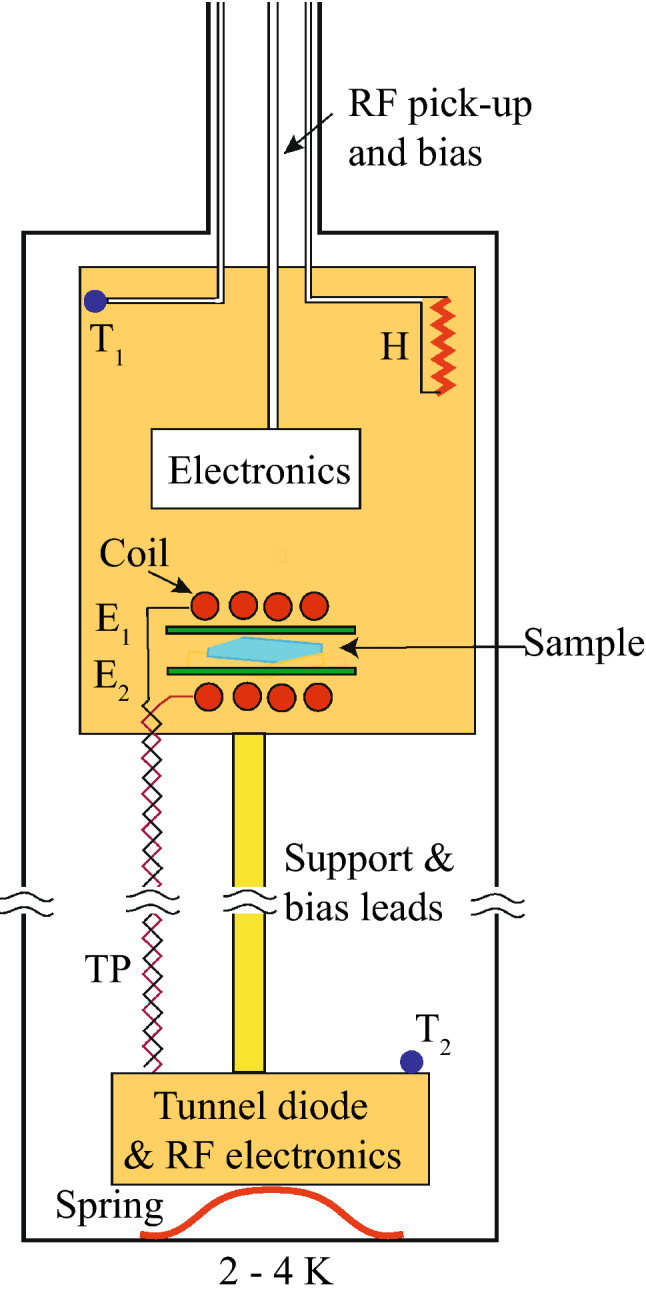
(Color online) Schematic representation of experimental apparatus (not to scale). The tunnel diode and associated bias circuit are located in the bottom block that is pressed against a cold plate at liquid helium temperatures. The support (yellow) maintains the pressure for the thermal contact. The resonant coil is located on a separate thermal block whose temperature is regulated by a feedback circuit feeding a heater H. The temperatures are measured with calibrated Cernox resistors^18,19^ T_1_ and T_2_. The separation between the sample block and the tunnel diode allows one to regulate the sample temepratures from 3.0 to 120 K. The twisted pair TP connects the tunnel diode to the resonant coil. Two electrodes (green) provide the electric field.

The ME effect was observed using an ultra-high sensitivity tunnel diode oscillator (TDO)^19^ operating at low temperatures to measure changes in the magnetic susceptibility of the order of parts per billion (ppb). A schematic representation of the oscillator system is shown in Fig. 2. The tunnel diode was maintained continuously at liquid helium temperatures for high sensitivity and high stability while the sample coil was thermally isolated from the TDO circuit and independently thermalized to a reference block whose temperature could be regulated between 3 and 120 K very precisely with a feedback loop. The sample was located inside a solenoidal radio frequency (RF) coil that oscillated at 15 MHz. The frequency was read by a precision frequency counter and digitized for sample averaging as the electric field was slowly modulated with a triangular wave shape having a period of 120 s. Data was also taken with a fixed electric field of 62 kV m^*−*1^as the temperature was swept slowly at a rate of 2.5 K hr^*−*1^ from 8.5 to 13.5 K. This temperature range was selected because the susceptibility studies of Georgopoulou *et al*. showed that in this temperature range the susceptibility is most affected by the antisymmetric exchange.

The change in susceptibility is determined from the fractional change in the oscillator frequency as a function of the temperature and field dependent magnetic susceptibility, *χ*(*E, T* ), from

1 $$\frac{\delta f}{f} = - 0.5\eta \chi^{^{\prime}} \left( {E,T} \right) + O\left( {Q^{ - 2} \chi^{^{\prime\prime}} } \right)$$

where *I*_o_ is the coil filling factor, Q the coil quality factor, and ${\chi }^{^{\prime}}$ and $\chi ^{{\prime \prime }}$ are the real and imaginary components of the magnetic susceptibility. The term in *Q*^*−*2^ can be neglected as Q is of the order of 10^3^ at low temperatures.

### Synthesis of [Fe_3_O(O_2_CPh)_6_(py)_3_]ClO_4_*·*py (Fe_3_)

Iron(III) perchlorate hydrate, benzoic acid, and pyridine were obtained from VWR and used as received. (Caution: Complexes between metal ions and organic ligands with perchlorate anions are potentially explosive. The compounds should be prepared in small amounts and handled with great care!) The synthesis of Fe_3_ was carried out according to the published procedure^14^.

Briefly, 1.44 g (*≈* 3.1 mmol) of Fe(ClO_4_)_3_*·*xH_2_O (x *≈* 6) was added with stirring to a solution of benzoic acid (1.22 g, 10.0 mmol) in 10 mL of pyridine. The resulting suspension was refluxed for 1.5 h and then allowed to cool to room temperature. After filtration, the filtrate was left to evaporate slowly on a warm heating plate (*≈* 35^*◦*^C), to afford X-ray quality crystals of Fe_3_. The reported unit cell was confirmed by single-crystal X-ray diffraction as reported in the Cambridge Crystallographic Data base (code QOPLUC). The compound forms in the centrosymmetric P6_3_/m space group and consists of trimers of Fe spins that are isolated from each as shown in Fig. 1.

## Results and discussion

The ultimate sensitivity of the TDO is determined by the shot noise of the device and the thermal stability of the diode. Robinson^23^ has shown that the shot noise in the current is given by

2 $$I_{n} = \sqrt {4eI_{0} B\left( {1 + f_{0} /f} \right)}$$

where *I*_*o*_ is the operating current, *B* is the post detection bandwidth and the corner frequency

*f*_*o*_* ≈* 10^3^ Hz.

The sensitivity to changes in diode conductance *G* is therefore

3 $$\frac{\delta G}{G} = \sqrt {4Fk_{B} TBQ/P_{0} }$$

for a power level ${P}_{0}$. With a noise factor $F\approx 10,$ this leads to a sensitivity of the order of 10^–9^ for an integration time ${B}^{-1}\approx 10 s.,$ which might be improved by averaging over a longer time.

The thermal variations were more troublesome (due to long internal time constants for the diode) and most of the fluctuations in the frequency of the oscillator with an empty cell were due to variations in temperature rather than shot noise. We therefore recorded data with the temperature for a very slow sweep with $\Delta T \approx 0.1\;{\text{K}} \cdot {\text{hr}}^{ - 1}$ (to keep the feedback circuit active) and modulated the applied electric field using a triangular amplitude as a function of time. The data were analyzed assuming a form

4 $$f_{k} = g\left( {T_{k} ,E_{k} } \right) + \xi_{k}$$

for each data point *f*_*k*_ where $g\left({T}_{k},{E}_{k}\right)$ is a model for the frequency response for an electric field ${E}_{k}$ and a temperature ${T}_{k}. \widetilde{g}$ is a fit to the data after averaging and ${\xi }_{k}$ is a random noise function.

The average $\widetilde{{g}_{k}}={C}_{0k}+{C}_{{T}_{k}}T+{C}_{{E}_{k}}E$ was found by minimizing $\sum_{l=k-n}^{k+n}[{f}_{l}-\widehat{{g}_{i}}({T}_{l},{E}_{l}){]}^{2}$ for n sweeps. Assuming $\widetilde{g}\left({T}_{l},{E}_{l}\right)=g\left({T}_{l},{E}_{l}\right)+\mathcal{O}(\frac{1}{\sqrt{n}})$ we have (using MKS units)

5 $$\widetilde{{g_{k} }}\left( {T_{k} ,E_{k} } \right) - \widetilde{{g_{k} }}\left( {T_{k} ,0} \right)] = - \frac{1}{2}\eta \left( {E\frac{{\partial \chi^{\prime}}}{\partial E}} \right)_{k}$$

**Figure 3:**
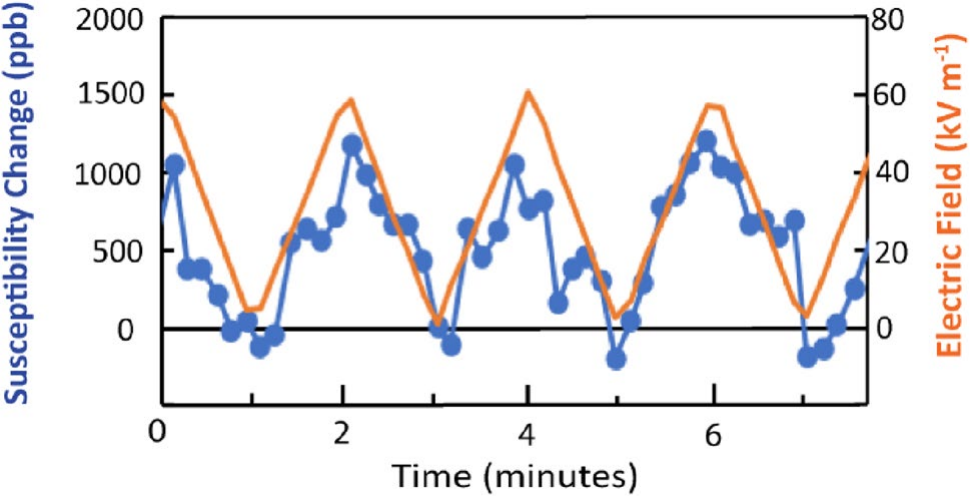
(Color online) Observed variation of magnetoelectric effect of the Fe_3_ trimer with a slow modulation of the applied electric field with peak field at 62 kV m^*−*1^. The electric field was aligned perpendicular to the RF magnetic field which was parallel to the c-axis of the crystal. The blue circles are the data points for the changes in magnetic susceptibility and the orange trace is the electric field.

Figure 3 clearly shows that the frequency response resulting from the susceptibility change is syn- chronous with the time dependence of the electric field. The amplitude of the observed ME effect

is indeed small at 16 ppb$\pm 3$ per kV m^*−*1^. This value is within the range estimated theoretically by Yu et al.^24^ who predicted a quadratic effect but for a different material, namely Mn_4_Te_4_ which has tetrahedral spin frustration, in contrast to the Fe_3_- trimer which has triangular frustration and thus could have a very different ME effect. The experiment was repeated for an empty cell but containing N-grease and no ME effect was observed.

The results presented here do not have the resolution to test that prediction. Nevertheless, the results can be compared with the observation of Boudalis *et al.*^15^ who found approximately a 1% effect for 10^9^ V m^*−*1^, or 10 ppb per kV m^*−*1^ which would be consistent with the results reported here for a linear effect. In order to obtain a better test the linear electric field dependence we would need to be able to apply an appreciably higher electric field, or modify the detector to obtain much higher sensitivity. Increasing the electric field to increase the magnitude of the effect is precluded because of voltage breakdown at small interfaces in the wiring at low temperatures, but improved sensitivity could be obtained by designing an ultra-high frequency (UHF) cavity resonator and that is being actively pursued. Conducting the experiments at much lower temperatures would run into problems with the long relaxation times at those temperatures.

**Figure 4:**
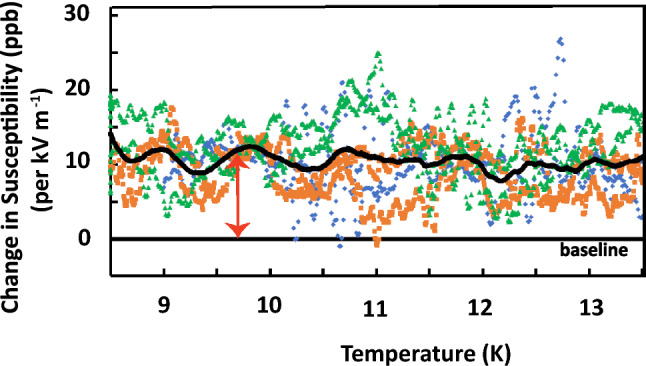
(Color online) Observed shift of magnetic susceptibility of the Fe_3_ trimer for a fixed applied electric field for several temperature sweeps. The blue, green and orange data points are the direct observations of the changes for four different sweeps with no averaging, and the solid black line is a filtered overall average as described in the text.

The data for a fixed electric field is shown in Fig. 4. While there is a detectable effect shown by the red arrow, the variation in noise amplitude from one temperature to another at a later time was quite large. We have therefore averaged the electric field induced changes over several temperature sweeps, and assuming the paramagnetic temperature dependence of Ref. 19, we find the average magnitude of the ME effect over this full temperature range to be 11 ppb$\pm 2$ per kV m^-1^. This value is consistent with the results of Boudalis et al.^15^.

Thus, we have observed electric field-induced changes in the magnetic susceptibility in Fe_3_, which is a direct observation of ME coupling. We note that the Fe_3_ compound forms in a centrosymmetric space group P6_3_/m^14^, whereas a ME coupling requires broken centrosymmetry. Thus any observed ME coupling indicates that centrosymmetry has become broken by the magnetic configuration of the Fe spins. Such spatial inversion symmetry breaking is predicted to occur in antiferromagnetic triangles like Fe_3_ via magnetic frustration and/or DM interactions^2,4,13,24^, both of which are present in this material. In general, the microscopic mechanisms by which magnetic configurations can induce electric dipoles in all materials involves (1) magnetostriction, e.g. distortion of the location of the charged ions in the lattice and/or (2) rearrangements of the electronic orbitals. These changes in the lattice and orbitals are driven by a need to minimize the magnetic energy at the expense of lattice distortion energy by modifying interactions such as magnetic exchange, single-ion anisotropy, or DM interactions.

## Conclusion

Studies of the influence of an applied electric field on the magnetic susceptibility of an Fe_3_ trimer, [Fe_3_O(O_2_CPh)_6_(py)_3_]ClO_4_*·*py, using a stabilized cryogenic tunnel diode oscillator, have revealed the existence of a small electrically-induced change in the magnetic susceptibility, of 11 ppb$\pm 2$ per kV m^*−*1^ for 8.5 < T < 13.5 K. The overall magnitude is in the range of the theoretical results by Yu et al.^22^*.* and is consistent with the change in ESR amplitude reported by Boudalis et al.^15^. It will be important to extend these studies to higher sensitivities, for example, by increasing the frequency of the oscillator to the 200–250 MHz range. This extension will also provide information about dynamics of the magnetic spin system and its dependence on the electric field.

## Data Availability

The datasets generated and/or analysed during the current study are available in the Cambridge Crystallographic Data Centre (CCDC) repository**,** and can be retrieved from the CCDC by referring to the entry code QOPLUC.
